# Breath Ethane Concentrations in Healthy Volunteers Correlate with a Systemic Marker of Lipid Peroxidation but Not with Omega-3 Fatty Acid Availability

**DOI:** 10.3390/metabo4030572

**Published:** 2014-07-08

**Authors:** Brian M. Ross, Iain Glen

**Affiliations:** 1 Division of Medical Sciences, Northern Ontario School of Medicine, Lakehead University, 955 Oliver Road, Thunder Bay, Ontario P7B5E1, Canada; 2 Highland Psychiatric Research Foundation, University of the Highlands and Islands, Inverness IV3 5SQ, UK

**Keywords:** oxidative stress, tocopherol, hydroperoxides, omega-3 fatty acid

## Abstract

Ethane in human breath derives from lipid peroxidation, specifically the reaction between omega-3 fatty acids and reactive oxygen species. It has been proposed to be a non-invasive marker of oxidative stress, a deleterious process which may play an important role in the pathophysiology of several common diseases. It is unclear, however, whether ethane concentration actually correlates with systemic oxidative stress or whether it is primarily a marker of airway biochemistry. To investigate this possibility the breath ethane concentrations in 24 healthy volunteers were compared to that of a systemic measure of oxidative stress, plasma hydroperoxides, as well as to blood concentrations of the lipophilic anti-oxidant vitamin E, and the abundance of omega-3 fatty acids. Breath ethane concentrations were significantly (*p* < 0.05) positively correlated with blood hydroperoxide concentrations (*r*_p_ = 0.60) and negatively with that of vitamin E (*r*_p_ = −0.65), but were not correlated with either the total omega-3 fatty acid concentration (*r*_p_ = −0.22) or that of any individual species of this fatty acid class. This data supports the hypothesis that breath ethane is a marker of systemic lipid peroxidation, as opposed to that of omega-3 fatty acid abundance.

## 1. Introduction

Oxidative stress is a deleterious process which occurs as a consequence of normal oxidative metabolism. It involves the generation of reactive oxygen species (ROS) such as the hydroxyl and superoxide radicals which react with other cellular constituents such as DNA, proteins and fatty acids [1]. These biomolecules become functionally altered as a result with subsequent damaging effects upon cellular function which, if severe enough, can lead to cell death. To combat the unwanted effects of this process oxidative stress is reduced by a number of different “anti-oxidants”. These include anti-oxidant chemicals, such as the tocopherols, which react preferentially with ROS thereby saving other cellular components, as well as detoxifying enzymes, such as superoxide dismutase, which catalyses the conversion of ROS into less toxic species. It is therefore the balance between the rate of ROS production and their removal by antioxidants that determines the degree of oxidative stress experienced by a cell [1]. 

Oxidative stress has been shown to be elevated in a variety of disease conditions including neurodegenerative disease, psychiatric illness, stroke, cancer, cardiac disease, and diabetes, and has been proposed to play a role in the pathophysiology of these conditions involved in both the initiation of disease (such as in cancer) and the resulting pathological changes (such as in diabetes) [2,3,4,5,6,7]. As such, the reduction and assessment of oxidative stress is viewed as being of significant clinical importance. The latter, monitoring, rarely involves the measurement of the ROS themselves since they are very short lived species. Instead assessment of oxidative stress is accomplished using a variety of indirect measures including testing for changes to anti-oxidant chemical and enzyme concentrations or by measuring the abundance of oxidation products which are formed in the reaction between ROS and cellular constituent [8,9]. In the case of fatty acids, reaction with ROS proceeds by a process known as lipid peroxidation which predominantly involves polyunsaturated fatty acids [1]. Specifically, ROS react with the fatty acid and oxygen to form a lipid peroxyl radical which either reacts with neighbouring fatty acids or is reduced to form a lipid hydroperoxide, compounds which are stable enough to be easily assayed in various tissues including blood [8,9]. Aside from the peroxides other compounds are also formed including isoprostanes, aldehydes and alkanes, several of which are volatile enough that they can be detected in breath [10].

One class of compounds, the alkanes, are produced as secondary products of the reaction between ROS and unsaturated fatty acids [1]. Specifically, lipid hydroperoxides via a process known as β-scission, give arise to ethane or pentane, the former from fatty acids which have their first carbon-carbon double bond 3 carbon atoms from the terminal “omega” carbon (omega-3 or n-3 fatty acids) and the latter from omega-6 (n-6) fatty acids [1]. Since pentane can be metabolized in the liver to pentanol the interpretation of altered breath pentane concentrations is more complex that of ethane which does not undergo such metabolism [11]. Ethane is also highly volatile resulting in it being predominantly excreted in the breath where it can be assayed using thermal desorption gas chromatography mass spectrometry or light spectroscopy, it being found in concentrations ranging from approximately 0.5 to 5 PPB by volume in the exhalant of healthy individuals [6,12]. Moreover breath ethane concentrations are increased in a variety of disorders including schizophrenia, attention deficit hyperactivity disorder, hepatic damage associated with alcohol misuse, hypertensive disorders of pregnancy, asthma, and obstructive pulmonary disease, findings which led the authors of each study to conclude that oxidative stress is increased in these conditions [6,12,13,14,15]. This conclusion has been questioned given that breath ethane concentrations were reported to be unrelated to protein carbonyl abundance, a measure of the effect of oxidative stress upon proteins [16]. It is also unclear as to whether changes in ethane concentrations are due to altered systemic lipid peroxidation or, alternatively, are largely pulmonary in origin. Indeed, in addition to pulmonary diseases, smoking tobacco products has variously been said to elevate ethane concentrations, presumably due to airway inflammation, but others have reported no effect [17,18]. Furthermore, since adding omega-3 fatty acids to the growth medium of cultured cells increases ethane concentrations in the culture “headspace”, the relative influence of omega-3 fatty acid availability and oxidative stress on breath ethane concentration is of interest [19]. For example, although it has been reported that increased breath ethane concentration in schizophrenia is not related to changes in omega-3 fatty acid concentrations , how varying degrees of oxidative stress influence this relationship is unknown [20]. To further investigate the relationship between breath ethane concentration and systemic oxidative stress we have correlated breath ethane concentrations with several systemic measures including the concentration of erythrocyte fatty acids, plasma hydroperoxides and the lipid soluble antioxidant vitamin E.

## 2. Methods

### 2.1. Human Participants

Twenty four subjects (13 female and 11 male) having a mean age of 38 ± 11 years were recruited by advertisement according to a protocol (2000–2014) approved by the NHS Highlands human ethics review board. After giving informed consent participants completed a health questionnaire. According to this no participants suffered from any acute illness or any chronic respiratory disorder according to a health questionnaire. 10 participants smoked tobacco products, with the smokers consuming between 1 and 30 cigarettes per day. 

### 2.2. Biological Sample Collection

Venous blood was collected from all subjects into a tube pre-treated with EDTA. The samples were kept on ice for no more than 60 min then spun at 1500 g_av_ for 15 min at 4 °C. The plasma layer and the buffy coat were separated off and the erythrocytes washed with an equal volume of 0.9% saline. Erythrocytes and plasma were stored at −80 °C prior to analysis. Breath samples were obtained from subjects using a syringe system (Markes International Ltd., UK) of volume 130 mL equipped with a disposable mouthpiece. The subject was instructed to exhale in one long breath into the syringe until they could no longer breathe out to collect the alveolar (end expired) air from the lungs as previously describe [6,12].

### 2.3. Analytical Procedures

All procedures have previously been described in detail. To quantify fatty acids erythrocytes were thawed on ice, lipids extracted and trans-esterified, and the resulting methyl esters quantified using gas chromatography [21]. Total plasma lipid hydroperoxides were measured by their ability to convert ferrous to ferric ion in the presence of xylenol orange (FOX assay) [22,23]. Vitamin E was quantified in plasma using high performance liquid chromatography [24]. The measured volume of expelled air was then analysed by thermal desorption gas chromatography and ethane quantified using mass spectrometry exactly as described [6]. The intra-assay variation (coefficient of variance) for ethane measurements was approximately 10%; intra-breath variation was approximately 18%. The limit of detection of the assay was approximately 0.1 PPBV.

## 3. Results

**Figure 1:**
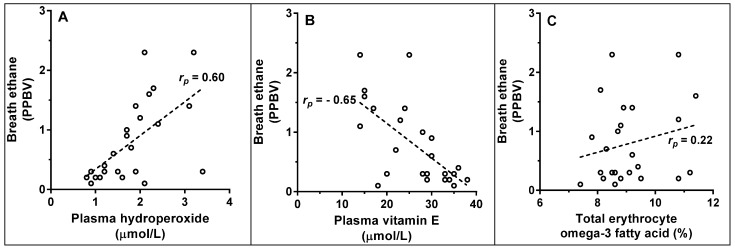
Breath ethane concentration was compared to concentrations of (**A**) hydroperoxides, (**B**) vitamin E, and (**C**) total omega-3 fatty acids in 24 healthy volunteers. The dotted line indicates the “best fit” linear regression line; the Pearson correlation coefficients are shown on the graphs.

The ethane concentration measured in the breath of 24 healthy volunteers was compared to that of blood hydroperoxides, vitamin E, total omega-3 fatty acids, age and number of cigarettes smoker per day of the same participants using regressional analysis. Multiple variable regressional analysis yield a statistically significant (*F*_5,18_ = 7.45; *p* < 0.001) R-squared value of 0.67 with vitamin E and hydroperoxides concentration contributing statistically significant influences upon the variance in breath ethane concentrations. Using individual regressional analysis revealed that ethane concentration was correlated to hydroperoxide concentration (*r*_p_ = 0.60), negatively correlated to vitamin E concentration (*r*_p_ = −0.65), and not significantly correlated to total omega-3 fatty acid concentration (*r*_p_ = −0.22; Figure 1), nor to the concentrations of individual omega-3 fatty acids (Table 1). Similarly, hydroperoxide concentration was not significantly correlated to total omega-3 fatty acid abundance (*r*_p_ = 0.32) but was significantly negatively correlated to vitamin E concentration (*r*_p_ = −0.43).

**Table 1:** Lack of correlation between breath ethane concentrations and various omega-3 fatty acid species.

Fatty acid	Mol %	Correlation with breath ethane
C18:3 n-3 (α-linolenic)	0.24 ± 0.05	−0.13
C18:4 n-3 (stearidonic)	0.20 ± 0.06	0.35
C20:3 n-3 (eicosatrienoic)	0.09 ± 0.05	0.11
C20:4 n-3 (eicosatetraenoic)	0.12 ± 0.05	−0.03
C20:5 n-3 (eicosapentaenoic)	0.96 ± 0.26	0.31
C22:5 n-3 (docosapentaenoic)	2.7 ± 0.41	0.33
C22:6 n-3 (docosahexaenoic)	4.5 ± 0.82	−0.16

For each fatty acid the mean ± standard deviation (relative concentration) in erythrocytes obtained from 24 healthy volunteers, along with the Pearson coefficient for the correlation with breath ethane, is shown. No correlations reached statistical significance.

## 4. Discussion

Our major finding is that breath ethane concentrations correlate with another measure of lipid peroxidation suggesting that ethane is a valid non-invasive measure of lipid peroxidation. Such a conclusion contradicts previous findings which found no correlation between breath ethane concentration and protein carbonyls, a measure of the oxidative damage of proteins [16]. A possible explanation for this discrepancy is that the present study correlated ethane with a measure of lipid peroxidation, not protein oxidation. Although the effects of oxidative stress on the two class of biomolecules are related, differential effects have been noted previously in other systems [25,26]. For example, supplementation of human subjects with omega-3 fatty acids increases lipid peroxidation but not the abundance of protein oxidation products [25]. Taken together the available evidence suggests that the assay of breath ethane concentrations can be used to assess the degree of lipid peroxidation but caution should be used if contemplating the use of ethane to estimate damage to other biomolecules such as proteins and DNA.

The presented data also indicate that ethane concentrations are, in healthy volunteers at least, indicative of systemic oxidative stress levels rather than being a measure of airway lipid peroxidation. This was evident even though a significant minority of participants smoked tobacco and hence might have been expected to exhibit some airway inflammation. While it cannot be ruled out that in other conditions involving airway inflammation that the source of ethane is predominantly pulmonary in origin (as has been suggested by others [16]), the data presented herein suggest that in persons who are not suffering from pulmonary disorders breath ethane is a measure of systemic lipid peroxidation and oxidative stress. Although such a conclusion needs to be confirmed by comparing ethane concentrations to other measures of lipid peroxidation such as conjugated dienes and malondialdehyde [1], the fact we found a negative correlation found between breath ethane and the circulating concentrations of vitamin E lends further support to ethane being a valid marker of systemic lipid peroxidation. This is consistent with the observations of others who have shown a negative relationship between tocopherols and hydroperoxides concentrations in healthy persons (although no relationship occurs in those with type 2 diabetes [27] who exhibit higher levels of oxidative stress), and that the tocopherols correlate more strongly with blood hydroperoxides concentrations than more hydrophilic antioxidants [28]. Since vitamin E has a dietary origin, our findings do suggest that breath ethane concentrations may be under some degree of dietary control via an effect upon the degree of oxidative stress.

The lack of correlation with erythrocyte omega-3 fatty acid concentrations in erythrocytes, however, suggests that dietary lipid intake does not exert a major influence on breath ethane concentration, particularly given that the distribution of fatty acid species in erythrocytes correlates with that found in other tissues. This extends a previous study performed in patients with schizophrenia [20]. Such data are dissimilar to in vitro findings in which supplementation of cell lines with omega-3 fatty acids increases ethane production [19]. Indeed it would be expected that at very low omega-3 concentrations ethane concentrations would fall. This discrepancy may relate to the more restricted range over which omega-3 fatty acids vary in the intact organism compared to that which can be achieved *in vitro*. As such our data suggest that over the normal physiological range, omega-3 fatty acid availability is not limiting on ethane production, but rather this is under control of the rate at which close to saturating amounts of these fatty acids can be oxidized by free radicals. Moreover, given that hydroperoxide concentrations did not correlate with omega-3 fatty acid abundance either makes it likely that such a lack of effect holds true over varying degrees of lipid peroxidation. We cannot rule out, however, that breath ethane concentrations may be influenced by fatty acid availability in conditions of significant dietary deficiency or supplementation, possibilities which will be addressed in a future study.

## 5. Conclusion

In summary, the data presented support the notion that breath ethane concentrations are correlated with another systemic marker of oxidative stress, but not with omega-3 fatty acid availability. Breath ethane is likely therefore a non-invasive measure of systemic oxidative stress in healthy individuals which is unlikely to be affected by changes in dietary fatty acid intake. Our findings may assist in the interpretation of breath ethane changes in disease conditions, *i.e*., that such a change is indeed due to altered oxidative stress. Furthermore, our data suggest further diagnostic and disease monitoring applications for breath alkane analysis, such as in the management of diabetes in which elevated oxidative stress may relate to worsened health outcomes.
